# Preliminary Characterization and Bioactivities of Some *Impatiens* L. Water-Soluble Polysaccharides

**DOI:** 10.3390/molecules23030631

**Published:** 2018-03-11

**Authors:** Katarzyna Szewczyk, Esther Marie Heise, Jakub P. Piwowarski

**Affiliations:** 1 Department of Pharmaceutical Botany, Medical University of Lublin, 1 Chodźki St., Lublin 20-093, Poland; 2 Department of Pharmaceutical Biology, Christian-Albrechts-University of Kiel, Gutenbergstrasse 76, D-24118 Kiel, Germany; mheise@pharmazie.uni-kiel.de; 3 Department of Pharmacognosy and Molecular Basis of Phytotherapy, Warsaw Medical University, 1 Banacha St., 02-097 Warsaw, Poland; jakub.piwowarski@wum.edu.pl

**Keywords:** *Impatiens*, polysaccharides, *Balsaminaceae*, antioxidant, immunostimulatory activity, cytotoxicity

## Abstract

Preliminary characterization and bioactivity of water-soluble polysaccharides from four *Impatiens* species—*I. glandulifera* Royle, *I. parviflora* DC., *I. balsamina* L., and I. *noli-tangere* L.—were investigated. The yields of polysaccharides range widely from 1.97% for *I. parviflora* roots to 18.63% for *I. balsamina* aerial parts. SEC (Size exclusion chromatography) chromatograms show that all samples contained a low molecular weight part that consisted of components of similar molecular weight. The aerial parts and roots of *I. balsamina*, and *I. glandulifera* aerial parts had considerable amounts of high molecular weight components up to 2.3 MDa. The sugar composition analysis revealed that *Impatiens* polysaccharides consisted primarily of galactose, arabinose, rhamnose, mannose, xylose, and glucose. All polysaccharide fractions, except for *I. parviflora* roots, also contain galacturonic acid. Moreover, in vitro bioactivity of obtained polysaccharides were evaluated. The antioxidant activity was evaluated on the basis of 2,2-diphenyl-1-picrylhydrazyl (DPPH) and 2,2-azino-bis-(3-ethyl-benzthia-6-sulfonic acid) (ABTS) radical scavenging assays. The highest antioxidant activity was obtained for *I. balsamina* aerial parts and *I. parviflora* roots. Among the tested fractions, only the polysaccharides from *I. glandulifera* aerial parts were able to significantly decrease the production of IL-8 by 32.7 ± 10.5%. The results suggest that *Impatiens* species can be considered as a new source of antioxidants.

## 1. Introduction

Plant polysaccharides have proven therapeutic activities such as anti-tumour, antiulcer, anti-inflammatory, wound healing, immunomodulatory, and anti-atherosclerotic [1,2]. It is believed that most of these effects are related to various parts of the immune system, and involved constituents of the innate immune system, e.g., the liberation of reactive oxygen species (ROS), and cytokines by granulocytes, dendritic cells, and macrophages [2,3]. Various polysaccharides derived from the plants demonstrated to interact specifically with pattern identification receptors on innate leukocytes; for example, scavenger receptors on leukocytes [1,4]. Moreover, most of non-digestible plant polysaccharides reduce blood pressure and cholesterol levels, and suppress the urinary markers of bone resorption [2,5]. There is also growing evidence that dietary carbohydrates may be helpful in brain function via the digestive tract due to the activation of parasympathetic nerve fibres or hormonal signalling [5,6,7].

*Impatiens glandulifera*, *I. noli-tangere* and *I. parviflora* are wild, invasive plants growing in Poland. These species have been shown to contain various groups of active compounds including, among others, phenolic acids [8], flavonoids [9,10], essential oils [11], naphthoquinones [12,13,14], fatty acids [13], steroids [15], and triterpenoid saponins [16]. *I. balsamina* contain also baccharane glycosides [17,18,19], coumarins [20] and anthocyanins [21]. However, to the best of our knowledge, there is only one paper published so far that has focused on the characterization of non-cellusosis polysaccharides of *I. parviflora* leaves [22]. Based on the significance of *Impatiens* species from a therapeutic activity and insufficient reports on the polysaccharides, the goal of the present work was to undertake a preliminary characterization of polysaccharides of aerial parts and roots of *I. glandulifera* Royle, *I. parviflora* DC. and *I. noli-tangere* L. Moreover, in vitro bioactivity of the *Impatiens* polysaccharides was evaluated. 

## 2. Results and Discussion

To obtain polysaccharide fractions exhaustive, multi-stage extraction was used. After the main extraction with hot water by sonication, deproteinization using the Savage reagent (2% isoamyl alcohol in chloroform) was done. Light-gray (*I. noli-tangere* and roots of *I. balsamina*) and light-beige (*I. glandulifera*, *I. parviflora* and aerial parts of *I. balsamina*) crude water-soluble polysaccharides were obtained.

The yields of polysaccharide fractions, which were related to the dried materials, are given in Table 1. These yields range widely from 1.97% for *I. parviflora* roots (IPR) to 18.63% for aerial parts of *I. balsamina* (IBH). High values were also obtained for *I. glandulifera* aerial parts (IGH; 8.32%) and roots (IGR; 7.74%). In contrast, Callaghan et al. [23] found that stem bases and roots of *I. glandulifera* contain 2.59% of soluble carbohydrates. The total yields of polysaccharide fractions from tested species (apart from *I. parviflora* roots) were also higher than those obtained from *I. parviflora* leaves using water extraction (3.0 wt%) [22]. Different yields of polysaccharides obtained by the cited authors and those in our study might be related to anatomic features of plant material and the harvest time.

The total neutral sugar content was determined by Dubois et al.’s [24] method. The highest values of neutral sugars contained aerial parts and roots of *I. balsamina*, 52.17% and 47.42% respectively. The aerial parts of *I. noli-tangere* (INH) contained comparable amounts of neutral sugars (47.96%). The obtained results were presented in Table 1.

The contents of uronic acids were the highest for *I. balsamina* aerial parts (8.94%) and roots (IBR; 7.52%) while the lowest for roots of *I. parviflora* (1.48%) and *I. noli-tangere* (INR; 2.81%). All water-soluble polysaccharides fractions contained also phenolic compounds. The highest quantity of total phenolics was found in the fraction from roots of *I. parviflora* (38.27 mg GAE/g DE). The uronic acids and total phenolics content are given in Table 1.

Based on the data in Table 1, it can be concluded that the monosaccharide compositions of polysaccharides from all examined species are quite similar, except for roots of *I. parviflora* and *I. noli-tangere*. They mainly consist of arabinose, rhamnose, galactose, mannose, xylose, and glucose, but in different amounts. Galactose was the predominant neutral sugar component, which was presented in all samples. All polysaccharide fractions, except for roots of *I. parviflora*, also contain galacturonic acid. The highest amounts of galacturonic acid were reported for aerial parts of *I. glandulifera* and *I. parviflora* (IPH), which contain 46.92 and 37.60 mol% of this acid respectively. Only roots of *I. parviflora* and *I. noli-tangere* do not contain glucuronic acid. According to Hromadkova et al. [22], the main monosaccharide components of *I. parviflora* leaves were glucose, galactose and arabinose, and these results are similar to those obtained in our study.

**Figure 1:**
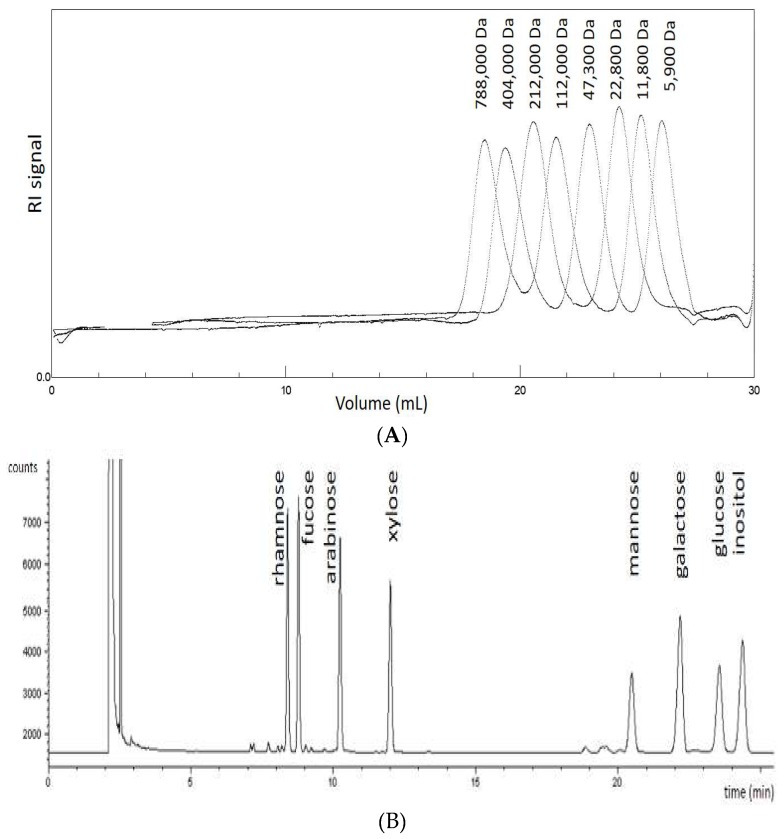
Size-exclusion chromatography (SEC) chromatograms of (**A**): pullalan standards, and (**B**): neutral sugars standards.

Size exclusion chromatography (SEC), using different pullulans with molecular masses of 788,000, 404,000, 212,000, 112,000, 47,300, 22,800, 11,800, 5900 Da as standards, was employed to establish the average M_W_ of the polysaccharide fractions. After adding buffer to the samples from the aerial parts of *I. parviflora*, *I. balsamina* and *I. noli-tangere*, and from the roots of *I. balsamina* and *I. glandulifera*, there remained an insoluble jelly sediment. That was filtered away prior to injection into the chromatographic system. These insoluble components could not be measured by SEC. After adding buffer to the sample from the aerial parts of *I. glandulifera,* brown flakes appeared, that were filtered away before injection. The chromatograms of pullalan standards and neutral sugars standards are shown in Figure 1.

**Figure 2:**
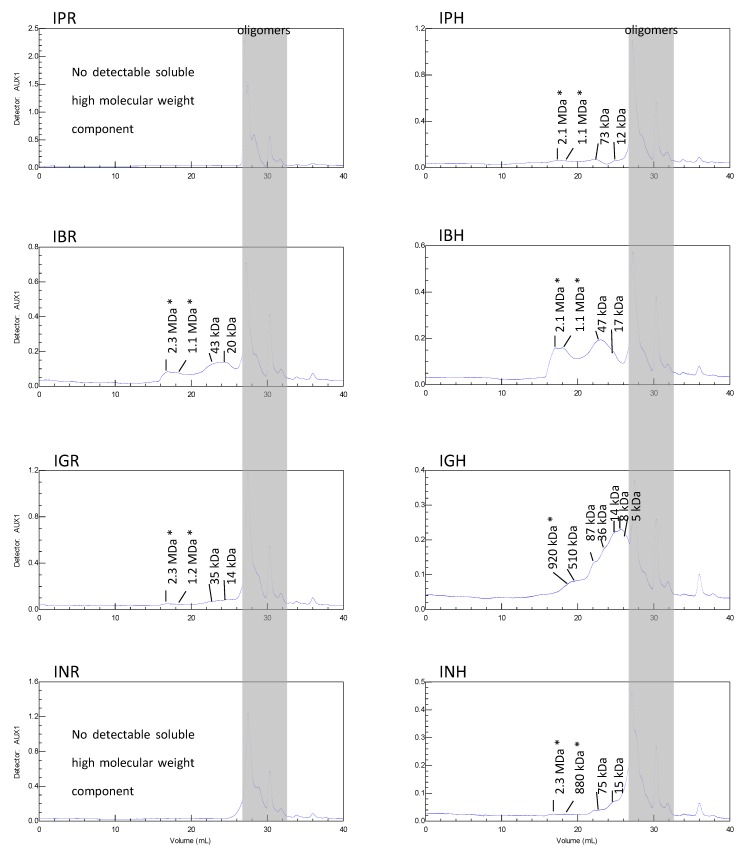
Size-exclusion chromatography (SEC) chromatograms of polysaccharides of *Impatiens* species. * The molecular weights out of calibration (the linear equation generated by the standard curve was y= −1.588ln(x) + 39.768; R^2^ = 0.9986).

SEC chromatograms show that all the samples contained a low molecular weight part (grey) that consisted of components of similar molecular weight. The samples from the aerial parts and roots of *I. balsamina*, and from the aerial parts of *I. glandulifera* had considerable amounts of high molecular weight components up to 2.3 MDa (the value was out of calibration and was calculated by using the calibration line) (Figure 2). Hromadkova et al. [22] reported that water-soluble polysaccharide fractions from the leaves of *I. parviflora* have a molecular weight ranging between 20 and 700 kDa. One of the obtained fraction contain also two smaller populations with M_p_ at 32 and 5 kDa. Similar low molecular weight populations were found in our study in the aerial parts of *I. glandulifera*.

A simple gel diffusion assay with all the samples was done [25]. A precipitation line shows up when a red dye called Yariv reagent is precipitating with arabinogalactan-proteins in the sample. The sharp orange precipitation line in gel diffusion assay with Yariv reagent shows the presence of arabinogalactan-proteins in the samples from the aerial parts and roots of *I. balsamina* and from *I. glandulifera* roots (weak), from the aerial parts of *I. glandulifera* and *I. noli-tangere*. These molecules are very interesting proteoglycans with only about 5–10% protein and a big branched carbohydrate part containing mainly galactose and arabinose. They are constituents of the cell wall, but can be linked to the plasma membrane via a GPI-anchor (a glycosylphosphatidylinositol anchor that can anchor molecules to the cell surface at the plasma membrane). They are involved in several functions for the plant (special growth processes, but also apoptosis) [26,27]. For arabinogalactans from *Echinacea,* a immunomodulation property was shown in vitro [28]. The aerial parts of *I. balsamina* and *I. glandulifera* seem to have a higher content of uronic acids. In general, these fractions are the most functional in bioassays. Both samples show arabinogalactan-protein neutral sugar distributions and form a precipitation line with Yariv reagent. 

Several recent studies indicate that water-soluble polysaccharides derived from the plant have biological activities, such as antioxidant [29,30], anticancer [31], anticoagulant [32], and immunological [2,3,6,29]. On this basis, the next stage of our research was to evaluate some biological properties of polysaccharide fractions obtained from *Impatiens* species. 

The antioxidant activity of water-soluble polysaccharide fractions was measured by two methods, the 2,2-diphenyl-1-picrylhydrazyl (DPPH) and 2,2-azino-bis-(3-ethyl-benzthia-6-sulfonic acid) (ABTS) radical scavenging assays. For all samples were found that the DPPH radical scavenging activity depended on concentration. At a dose of 4.0 mg/mL, the DPPH scavenging abilities were the highest for the aerial parts of *I. balsamina* (84.40%), *I. parviflora* (81.25%) and *I. glandulifera* (80.18%), and for roots of *I. parviflora* (81.95%), and these values were close to that of ascorbic acid (98.81%) and Trolox (96.99%). The EC_50_ values for scavenging DPPH radicals are shown in Table 2. The ABTS scavenging ability decreased in the following order: IBH > IPR > IPH > IGH > IBR > IGR > INH > INR. The highest antioxidant activity tested using ABTS· was obtained for the polysaccharide fractions from the aerial parts of *I. balsamina* and roots of *I. parviflora* (EC_50_ 0.32 and 0.38 mg/mL of dry extract, respectively). Taking into account antiradical activity, it may be concluded that this activity is dependent on part of plant, but also on molecular weight and monosaccharide composition. Previous studies suggest that polysaccharides with low molecular weight have stronger antiradical activities [33]. The data suggested that the content of uronic acids was an effective indicator to antioxidant activity of the samples. Besides, the previous literature assumes that the polysaccharide fractions with a low ratio of glucose have higher antioxidant activity [34]. Furthermore, the DPPH and ABTS radical abilities of obtained fractions might be associated to the presence of other classes of compounds with antioxidant activities. Although the polysaccharide fractions from *Impatiens* species contained a large percentage of sugars, they also had a smaller amount of antioxidants from other chemical groups. In general, good sources of antiradicals were the aerial parts of *I. balsamina* and roots of *I. parviflora*.

**Figure 3:**
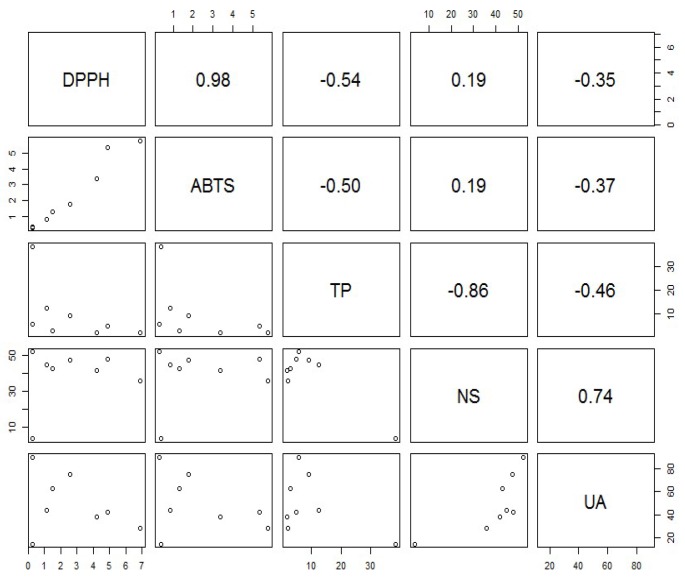
Pearson’s correlation coefficients between antioxidant activity (DPPH, ABTS) and total phenols (TP), neutral sugars (NS) and uronic acids (UA) content.

As can be seen from Figure 3. The results are highly intercorrelated between the assay methods (r = 0.98). However, the results are weakly correlated with total phenolic content. The correlation coefficient equals to −0.54 (DPPH) and −0.504 (ABTS). Such a correlation cannot be considered as significant in standard correlation significance test (*p* = 0.17 and *p* = 0.20, respectively). Low correlation was observed with neutral sugars content and this can be considered as significant (*p* = 0.66). 

None of the tested compounds expressed a cytotoxic effect towards neutrophils after 24 h incubation (Data not shown). 

**Figure 4:**
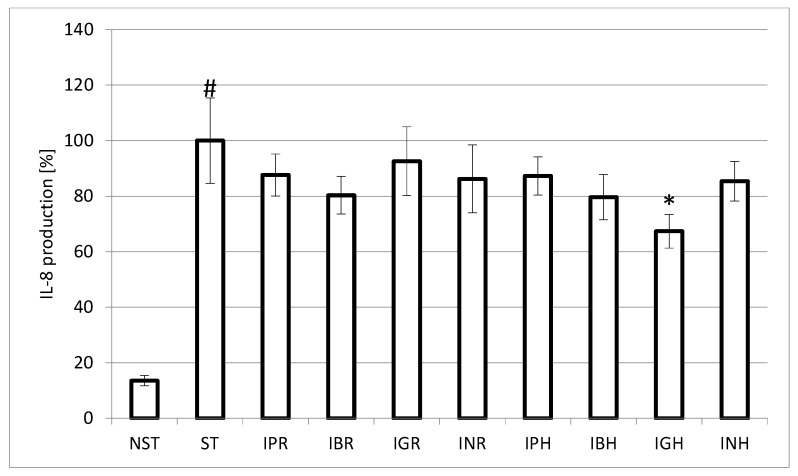
Effect of polysaccharides of *Impatiens* L. at the concentration of 50 μg/mL on interleukin-8 (IL-8) production by lipopolysacharide (LPS) stimulated neutrophils. Data were expressed as mean ± SEM of three separate experiments performed with neutrophils isolated from independent donors assayed in duplicate. * *p* < 0.05 versus stimulated control. #- statistically significant (*p* < 0.001) versus non-stimulated control; ST- stimulated control; NST- non-stimulated control.

Stimulation of neutrophils with LPS (lipopolysacharide) for 24 h led to significant induction of interleukin-8 (IL-8) production. Among the tested fractions, only the water-soluble polysaccharides from the aerial parts of *I. glandulifera* were able to significantly decrease the production of IL-8 by 32.7 ± 10.5%. The obtained results (Figure 4) suggested that polysaccharides were composed of high amounts of galactose, arabinose and rhamnose have potent strong immunostimulatory activity, and is consistent with the previous studies [32,35]. Other polysaccharide fractions in our study did not show any inhibition. 

Our findings suggest that polysaccharide fractions from studied *Impatiens* species contain similar monosaccharide composition to already reported in *Panax ginseng*, which are used in the medicine and food industry [36]. The high content and composition of obtained polysaccharides explain the traditional use of examined plants for the treatment of burns and would healing [37]. Furthermore, our results suggest that polysaccharides from *Impatiens* species are able to protect the human body from free radicals and might be good sources of natural antioxidants.

However, more studies are required on the fractionate *Impatiens* polysaccharides into homogeneous fractions, and further analyses should be done to better characterize each fraction, and evaluate their bioactivities and structure—activity relationship.

## 3. Materials and Methods 

### 3.1. Materials and Reagents

The plants (aerial parts and roots) were collected in July 2015. *I. balsamina* L. (no. IB-0715) was collected in the UMCS Botanic Garden, which is a part of Maria Curie Sklodowska University in Lublin, Poland at an altitude of 197 m a.m.s.l. (coordinates 51°08′41′′ N; 22°18′17′′ E). *I. glandulifera* Royle (no. IG-0715) was collected in Józefów near Biłgoraj (Józefów, Poland) at an altitude of 243 m a.m.s.l. (coordinates 50°28′54 N; 23°2′10′ E. *I. noli-tangere* L. (no. INT-0715) and *I. parviflora* DC. (no. IP-0715) were gathered in Zalesie Górne near Warsaw (Poland) at an altitude of 115 and 108 m a.m.s.l. (coordinates 52°2′16′′ N; 21°1′55′′ E and 52°2′2′′ N; 21°1′55′′ E respectively). Voucher specimens were deposited in the Department of Pharmaceutical Botany, Faculty of Pharmacy, Medical University of Lublin (Lublin, Poland). Plants were identified by Prof. Tadeusz Krzaczek.

All chemical reagents used in the experiment were purchased from various commercial suppliers and were of the highest purity available. 2,2-diphenyl-1-picrylhydrazyl (DPPH), 2,2-azino-bis-(3-ethyl-benzthia-6-sulfonic acid) (ABTS), (±)-6-hydroxy-2,5,7,8-tetramethylchromane-2-carboxylic acid (Trolox), ascorbic acid, 3-phenylphenol, gallic acid, d-glucose, d-galacturonic acid, were purchased from Sigma-Aldrich (St. Louis, MO, USA). l-glutamine, fetal bovine serum (FBS), 4-(2-hydroxyethyl)-1piperazineethanesulfonic acid (HEPES) and Roswell Park Memorial Institute (RPMI) 1640 medium were purchased from Sigma-Aldrich GmbH (Steinheim, Germany). LPS (lipopolysacharide) was purchased from Merck Millipore (Billerica, MA, USA). Propidium iodide was purchased from BD Biosciences (San Diego, CA, USA). All substances used were of >95% purity. Phosphate-buffered saline (PBS) was purchased from Gibco (Carlsbad, CA, USA).

### 3.2. Extraction of Polysaccharides

The powdered aerial parts and roots of examined species (50 g) were macerated two times with 99.8% ethanol (500 mL each time) for 24 h at room temperature to eliminate coloured materials and low molecular weight compounds. The obtained, after the supernatants removing, residues were extracted twice with ethanol (500 mL each time) in ultrasonic bath (InterSonic IS-4, Olsztyn, Poland) at room temperature for 30 min (180 W; 25 kHz ± 5%). Next, the dried residues were macerated two times with hot (80 °C) demineralized water (500 mL) by shaking for 3 h. After the supernatants removing, the residues were extracted twice for 30 min with demineralized water under sonication (180 W; 25 kHz ± 5%) at 80 °C. The combined solutions were concentrated under vacuum to 10 mL and purified by deproteinization using the Savage reagent (2% isoamyl alcohol in chloroform). Consequently, the polysaccharides were precipitated with frozen 99.8% ethanol at a ratio of 1:25 (*w*/*v*). Then, they were kept 12 h in the refrigerator at 4 °C. The crude polysaccharides were collected by centrifugation (8000 rpm, 20 min) and washed with acetone, and then lyophilized in a Free Zone 1 apparatus (Labconco, Kansas City, KS, USA). The percentage polysaccharide yield (%) was calculated as follows:

Yield (%·*w*/*w*) = weight of extracted polysaccharides/weight of dried plant material × 100

### 3.3. Chemical Composition Analysis

The total neutral sugar content was determined by Dubois et al. [24] method using d-glucose as a standard at 490 nm. A phenol-sulfuric acid method is based on the regression equation of glucose standard curve. The standard curve was prepared by analysing glucose calibration solutions at concentration ranging from 5 to 50 µg/mL. The regression equation between absorbance (y) and glucose concentration (x; µg/mL) found was y = 0.0167x + 0.1202, R^2^ = 0.9929. The total uronic acid content was determined using colorimetrically assay with 3-phenylphenol at 525 nm [38]. In this test d-galacturonic acid was applied as a standard. The phenol content was evaluated using the Singleton and Rossi [39] colorimetric procedure with some modifications. The absorbance was evaluated at 660 nm (Spectrophotometer UV-VIS, Thermo Evolution 300, Madison, WI, USA) and the results were expressed as mg of gallic acid equivalent (GAE) per 1 g of dry extract (DE). 

### 3.4. Monosaccharide Composition Analysis

To establish the monosaccharide composition of the polysaccharide fractions, the 10 mg of a sample was hydrolyzed with trifluoroacetic acid (2.0 mol/L) at 121 °C for 1 h. After evaporation of trifluoroacetic acid, monosaccharides were transformed to alditol acetates by reduction and acetylation as described by Blakeney et al. [40] and analyzed by gas liquid chromatography (GLC) on a fused silica capillary column (Optima-OV 225–0.25 µm, 25 m, i.d. 0.25 mm, Macherey-Nagel, Düren, Germany) as described by Göllner et al. [41]. For quantitative analysis, a defined amount of *myo*-inositol was added to the fractions as an internal standard. Monosaccharide standards and myo-inositol were run in advance to determine response factors for flame ionization detector (FID). For detection of uronic acids, after methanolysis the liberated methyl glycosides of the arabinogalactan protein (AGP) were transformed into the corresponding trimethyl silyl ethers (TMS-derivates) and analyzed by GLC [42,43].

### 3.5. Size Exclusion Chromatography (SEC)

The molecular masses of the samples were determined by size exclusion chromatography on two PL aquagel-OH MIXED M 8 µm 300 × 7.5 mm (with a range of 1 to 500 kDa) from Agilent and one PL-aquagel-OH MIXED H 8 µm 300 × 7.5 mm (with a range of 6 to 10,000 kDa) from Agilent columns in series (temperature 35 °C, Polymer Laboratories, Darmstadt, Germany). The samples were eluted with NaNO_3_ (0.85%; 0.1 M), NaH_2_PO_4_ (0.12%; 0.01 M) and NaN_3_ (0.05%) pH 7 buffer at a flow rate of 1.0 mL/min. The total run time was 40 min. The size of the components in the sample had to be calculated by their retention time. Different pullulans with molecular masses of 788,000, 404,000, 212,000, 112,000, 47,300, 22,800, 11,800, 5900 Da (PL Polysaccharide Standard Kit, Polymer Laboratories, Church Stretton, UK) were used as standards to establish the hydrodynamic volume. The detector was a refractive index detector (Polymer Laboratories).

### 3.6. Reactivity with Yariv Reagent

The reactivity with Yariv reagent was determined by using a simple gel diffusion test [25]. Five cavities were stamped in an autoclaved agarose-gel (1% agarose in 10 mM Tris-HCl-buffer, pH 7.3 with 0.9% NaCl and 1 mM CaCl_2_). The middle cavity was filled with Yariv-solution (c = 1 mg/mL), the four surrounding cavities with a solution of the polysaccharides (c = 50 mg/mL, 2 plates, samples 1–8). The characteristic positive reaction for the presence of AGPs was a sharp precipitation line between the Yariv-reagent cavity and the cavity of the sample after one day at room temperature.

### 3.7. Antioxidant Activity

To establish the antioxidant potential of water-soluble polysaccharide fractions two methods were applied. 2.2-diphenyl-1-picryl-hydrazyl (DPPH) free radical scavenging activity was evaluated in accordance with the Brand–Williams et al. [44] procedure. The changes in colour from deep-violet to light-yellow were evaluated at 515 nm in a UV/visible light spectrophotometer. 

The antioxidant activity was also evaluated using 2,2′-azinobis[3-ethylbenzthiazoline]-6-sulfonic acid (ABTS) decolourization assay [45]. The absorbance was measured at 734 nm, at the beginning and after 5 min of reaction. The ability of the polysaccharides to quench the ABTS free radical was determined according to equation: 

Scavenging% = [(A_C_-A_P_)/A_C_] × 100, where A_C_ is absorbance of control (solvent instead polysaccharides) at 0 min; A_P_—absorbance of polysaccharide fraction after 5 min.

The various concentrations of the water-soluble polysaccharides (0.25, 0.5, 1.0, 2.0, 4.0 mg/mL) were used. Both antioxidant activities were expressed as an efficient concentration EC_50_, the polysaccharide solution concentration providing 50% of the activity in a dose-dependent manner. Ascorbic acid and Trolox were used as a positive control.

### 3.8. Neutrophils Isolation

Peripheral venous blood was collected from healthy human donors (20–35 years old) in the Warsaw Blood Donation Centre. They did not smoke or take any medication. Donors were clinically confirmed to be healthy, and a routine laboratory tests demonstrated values within the normal range. The test corresponded to the rules of the Declaration of Helsinki. Neutrophils were isolated with a standard method [46] of dextran sedimentation and subsequent hypotonic lysis of erythrocytes and centrifugation in a Ficoll Hypaque gradient. The purity of neutrophils was >97%, and viability measured by trypan blue exclusion was >98%. 

### 3.9. Cytotoxicity

Cytotoxicity was established using standard flow cytometric method with propidium iodide (PI) staining. Neutrophils (3.5 × 10^5^ cells/mL) were cultured in a 24-well plate in RPMI 1640 medium with 10% FBS, 10 mM HEPES, and 2 mM l-glutamine for 18 h at 37 °C with 5% CO_2_ in presence of compounds at concentration of 50 μg/mL. After 18 h the neutrophils were harvested and centrifuged (2000 RPM; 10 min; 4 °C), washed once with cold PBS and resuspended in 500 μL of PBS. 5 μL of PI (50 μg/mL) solution was added to the cell suspension. After 15 min of incubation in darkness at room temperature cells were analyzed by BD FACS Calibur flow cytometer (BD Biosciences, San Jose, CA, USA), 10,000 events were recorded per sample. Cells that displayed high permeability to PI were expressed as a percentage of PI(+) cells.

### 3.10. IL-8 Production

Neutrophils (3.5 × 10^5^ cells/mL) were cultured in a 24-well plates in RPMI 1640 medium with 10% FBS, 10 mM HEPES, and 2 mM l-glutamine in the absence or presence of LPS (100 ng/mL) for 18 h at 37 °C with 5% CO_2_ in the absence or presence of compounds at final concentrations of 50 μg/mL added to 0.5 mL of cell suspension 1 h before the stimuli. After 18 h the neutrophils were harvested and centrifuged (2000 RPM; 10 min; 4 °C). The amount of released IL-8 into cell supernatants was measured by enzyme-linked immunosorbent assay (ELISA) following the manufacturer’s instructions (R&D Systems, Minneapolis, MN, USA).

### 3.11. Statistical Analysis

The results were presented as mean values ± SEM of the indicated number of experiments. Statistical significance of differences between means was determined by one-way ANOVA with Tukey’s post hoc tests. Results with *p*-value < 0.05 were considered statistically significant. All analyses were performed using Statistica 12 software.

## 4. Conclusions

The purpose of the study was to estimate and compare the chemical composition and biological activity of water-soluble polysaccharides from the aerial parts and roots of *I. parviflora*, *I. balsamina*, *I. glandulifera* and *I. noli-tangere*. Our findings demonstrated that the contents of neutral sugars, uronic acids, total phenols and monosaccharides were different from each other. The predominant neutral sugar component in all samples was galactose. A simple gel diffusion assay with Yariv reagent shows the presence of arabinogalactan-proteins in the samples from the aerial parts and roots of *I. balsamina*, roots of *I. glandulifera*, the aerial parts of *I. glandulifera* and *I. noli-tangere*. 

In vitro studies revealed that the water-soluble polysaccharide fractions from the aerial parts of *I. balsamina* and roots of *I. parviflora* might be a good source of antiradicals. Moreover, polysaccharides from the aerial parts of *I. glandulifera* were able to significantly decrease the production of IL-8 by 32.7 ± 10.5%.

To the best of our knowledge, this study represents the first report of the characterization and bioactivity of the water-soluble polysaccharides from different *Impatiens* species.

## Figures and Tables

**Table 1:** Characterization of a water-soluble polysaccharide fractions isolated from the aerial parts and roots of *I. parviflora*, *I. balsamina*, *I. glandulifera* and *I. noli-tangere*.

		IPR	IPH	IBR	IBH	IGR	IGH	INR	INH
**Yield** (% *w*/*w*)		1.97	4.30	6.73	18.63	7.74	8.32	4.30	4.72
**Neutral sugars** (%)		3.68 ± 0.03	44.96 ± 0.13	47.42 ± 0.31	52.17 ± 0.82	41.96 ± 0.15	43.06 ± 1.04	36.03 ± 0.10	47.96 ± 0.15
**Total phenolics** (mg GAE/g DE)		38.27 ± 0.54	12.53 ± 0.42	9.21 ± 0.13	5.81 ± 0.11	1.93 ± 0.06	3.02 ± 0.08	2.14 ± 0.11	5.03 ± 0.24
**Uronic acids** (%)		1.48 ± 0.02	4.38 ± 0.06	7.52 ± 0.23	8.94 ± 0.62	3.78 ± 0.15	6.83 ± 0.11	2.81 ± 0.06	4.23 ± 0.57
**Contents of the sugar** (mol %)	**Gal**	0.15	7.80	10.19	12.31	6.54	11.86	2.28	9.73
	**GalA**	n.d.	10.97	37.60	52.14	9.92	46.92	5.74	5.22
	**Ara**	n.d.	2.53	3.29	2.91	2.53	5.70	0.89	5.19
	**GlcA**	n.d.	7.17	15.29	17.71	6.69	10.03	n.d.	6.69
	**Man**	n.d.	3.04	3.80	5.62	2.74	2.43	1.22	3.34
	**Glc**	0.30	0.61	1.06	0.91	1.22	3.19	6.69	1.52
	**Rha**	n.d.	0.42	0.83	0.83	0.42	1.39	0.42	0.42
	**Xyl**	n.d.	0.13	0.13	0.25	0.25	0.25	0.38	0.25
	**Fuc**	n.d.	0.30	0.30	0.61	0.15	0.15	n.d.	0.30

IPR, *I. parviflora* roots; IPH, *I. parviflora* aerial parts; IBR, *I. balsamina* roots; IBH, *I. balsamina* aerial parts; IGR, *I. glandulifera* roots; IGH, *I. glandulifera* aerial parts; INR, *I. noli-tangere* roots; INH, *I. noli-tangere* aerial parts; Gal, galactose; GalA, galacturonic acid; Ara, arabinose; GlcA, glucuronic acid; Man, mannose; Glc, glucose; Rha, rhamnose; Xyl, xylose; Fuc, fucose; content of the sugars is expressed in mol % related to the total content of the monosaccharides; % w/w, yield related to the dried plant material; the total phenolic content is expressed as gallic acid equivalents; n.d., not detected.

**Table 2:** The antioxidant activity—DPPH and free radicals scavenging ability (ABTS) of polysaccharide fractions of the *Impatiens* species. The results are expressed as EC_50_ in mg/mL of DE (dry extract). Ascorbic acid (AA) and Trolox were used as the positive control. Each value is the mean ± SD (*n* = 3).

	IPR	IBR	IGR	INR	IPH	IBH	IGH	INH	AA	Trolox
**DPPH**	0.27 ± 0.02	2.55 ± 0.05	4.22 ± 0.06	6.89 ± 0.18	1.13 ± 0.02	0.24 ± 0.01	1.49 ± 0.01	4.85 ± 0.13	0.11 ± 0.01	0.08 ± 0.01
**ABTS**	0.38 ± 0.02	1.78 ± 0.03	3.37 ± 0.10	5.75 ± 0.19	0.84 ± 0.03	0.32 ± 0.02	1.30 ± 0.01	5.31 ± 0.13	0.21 ± 0.01	0.24 ± 0.02
